# Alzheimer and vascular brain diseases: Focal and diffuse subforms

**DOI:** 10.1590/1980-57642015DN93000015

**Published:** 2015

**Authors:** Eliasz Engelhardt, Lea T. Grinberg

**Affiliations:** 1 Full Professor (retired), Cognitive and Behavioral Neurology Unit - Institute of Neurology / Institute of Psychiatry - Federal University of Rio de Janeiro (UFRJ), Rio de Janeiro RJ, Brazil.; 2 Assistant Professor, Department of Neurology and Pathology, University of California, San Francisco, San Francisco, USA. 33Professor, PhD, Department of Pathology, School of Medicine, University of Sao Paulo, Sao Paulo SP, Brazil.; 3 Professor, PhD, Department of Pathology, School of Medicine, University of Sao Paulo, Sao Paulo SP, Brazil.

**Keywords:** Alzheimer, brain vascular disease, arteriosclerosis, vascular subtypes, Vascular Cognitive Impairment

## Abstract

Alois Alzheimer is best known for his description of the pre-senile neurodegenerative disease named after him. However, his previous interest in vascular brain diseases, underlying cognitive and behavioral changes, was very strong. Besides describing the Arteriosclerotic atrophy of the brain and the arteriosclerotic subtype of Senile dementia which he viewed as main forms of vascular brain diseases, he also identified and described a series of conditions he considered subforms. These may be divided, as suggested by the authors of the present paper, into 3 groups: gliosis and sclerosis, subcortical atrophies, and apoplectic. The subforms of the three groups present characteristic neuropathological features and clinical, cognitive and behavioral manifestations. These provide the basis, together with part of the main forms, for the contemporary condition known as Vascular Cognitive Impairment.

## Abstract (pt)

Alois Alzheimer é conhecido principalmente pela descrição de uma doença neurodegenerative pré-senil, que recebeu seu nome. Entretanto, previamente, seu interesse em doenças vasculares cerebrais, subjacentes a desordens cognitivas e comportamentais, foi muito forte. Além de descrever a Atrofia arteriosclerótica do cérebro e o subtipo arteriosclerótico da Demência senil, vistas por ele como formas principais de doenças vasculares cerebrais, ele identificou e descreveu uma série de condições que considerou como subformas. Estas podem ser divididas, como sugestão dos autores do presente artigo, em tres grupos: gliose e esclerose, atrofias subcorticais e apoplética. As subformas dos tres grupos apresentam aspectos neuropatológicas e manifestações clínicas, cognitivas e comportamentais, características. Estas forneceram a fonte, juntamente com parte das formas principais, à condição contemporânea conhecida como Comprometimento Cognitivo Vascular.

## INTRODUCTION

Aloysius [Alois] Alzheimer (1864-1915), psychiatrist and neuropathologist, became renowned for his description of a new disease that carries his name.^1^ However, his previous remarkable studies on brain vascular disorders underlying cognitive, behavioral and neurological manifestations, became forgotten. He intensively studied the subject, resulting in important conferences and lectures as well as in a few papers on the theme, published between 1894 and 1902. These studies contributed to establish key knowledge on what has become incorporated into the present status on the subject – the Vascular Cognitive Impairment spectrum.^2,3^

Besides the two main dementia forms, Arteriosclerotic atrophy of the brain (*arteriosklerotische Atrophie des Gehirns*) and Senile dementia (*senile Demenz*), both examined by the authors in previous papers,^2,3^ Alzheimer identified and described a series of conditions (diseases) related to atheromatous vascular degeneration of the brain arteries which he designated "subforms". He was able to distinguish these processes according to their location and spread of the brain changes, manifested by cortical and/or subcortical clinical symptoms.^4-9^ Besides the cases considered as typical subforms, he also mentioned the presence of mixed cases, mainly of vascular nature or more rarely, combined with syphilitic pathology, sometimes hampering the interpretation of a given case.^9^

The vascular subforms described and named by Alzheimer may be divided, here as proposed by the authors of the present paper, into 3 groups: gliosis and sclerosis, subcortical atrophies, and apoplectic. These subforms may also be regarded as focal and diffuse conditions (Table).

**Table:** Alzheimer and vascular brain diseases (Alzheimer [1894-1902]).^4-9^

**Main forms**
Arteriosclerotic atrophy of the brain (*arteriosklerotische Atrophie des Gehirns*) (Alzheime , 1894, 1902)^4,9^
Senile dementia (*senile Demenz*) (Alzheime , 1898, 1902)^7,9^
**Subforms **
**gliosis and sclerosis group **
•Perivascula gliosis (*perivasculäre Gliose*) (Alzheime , 1896, 1897, 1899, 1902)^5,6,8,9^
•Perivascula sclerosis (*perivasculäre Sklerose*) (Alzheime , 1899)^8^
•Perivascula gliosis of the cerebral cortex (*perivasculäre Gliose de Hirnrinde*) (Alzheime , 1898)^7^
•Perivascula sclerosis of the cerebral cortex (*perivasculäre Skle­rose de Hirnrinde*) (Alzheime , 1898)^7^
•Senile sclerosis of the cerebral cortex (*senile Sklerose de Hirn­rinde*) (Alzheime , 1899)^8^
•Senile cortical sclerosis [atrophy] (*senile Rindenverödung*) (Al­zheime , 1902)^9^
**subcortical atrophy group**
•Arteriosclerotic brain degeneration (*arteriosklerotische Hirnde­generation*) (Alzheime , 1898, 1899)^7,8^ (described by Alzheime and Binswange)
•Chronic progressive subcortical encephalitis (*Encephalitis sub­corticalis chronica progressiva*) •(Binswange , 1894)^17^ (Alzheime , 1898, 1902)^7,9^
•Arteriosclerotic atrophy of the hemispheric white matte (*arteri­osklerotische Atrophie des Hemisphärenmarks*) (Alzheime , 1899)^8^
**apoplectic group**
•Apoplectic dementia (*Dementia apoplectica* ) (Alzheime , 1898)^7^ [or]
•Post-apoplectic dementia (*Dementia post apoplexiam* ) (Alzheime , 1902)^9^

Hereunder, the characteristics of these subforms will be considered as condensed excerpts of Alzheimer's descriptions, relevant for the present approach, extracted and brought together from his several writings, and excluding conditions extraneous to the present scope (Box 1, Box 2, and Box 3).

**Box 1:** The gliosis and sclerosis group, comprising subforms described and named by Alzheimer (condensed excerpts).

**Perivascular gliosis** (*perivasculäre Gliose*). Characterized by severe arteriosclerosis of brain vessels and marked glial proliferation, with disseminated focal lesions in the cortex and white matter, restricted to one or more gyri, or single brain lobes, and expressed clinically as cortical focal disease (hemianopia, aphasia, cortical deafness, hemiplegia). Mild retraction or greater coarseness of a given gyral surface, on macroscopic neuropathology, and focal lesions with degeneration and loss of ganglion cells [neurons] and of myelinated fibers, on microscopy^5,6,9^.
**Perivascular sclerosis** (*perivasculäre Sklerose*). Previous description reiterated, with the assumption that it would be the cause of Perivascular sclerosis, due to stenosis of one larger arterial branch, with restriction of the necessary nutrition of the territory it supplied, and followed by the above-mentioned changes^8^.
**Perivascular gliosis of the cerebral cortex***(perivasculäre Gliose* *der Hirnrinde*). Characterized by arteriosclerotic focal lesions and marked glia proliferation, unevenly distributed throughout the entire cerebral cortex. Small depressions on the cortical surface, with finely or coarsely granulated texture, on gross examination. Individual foci with wedge-shaped appearance, the broader side facing the surface, and the peak reaching the 4^th^ or 5^th^ cortical layer, on microscopy. Older foci evolving occasionally to small softenings, with a degenerated vessel in its central part, where the ganglion cells appeared destroyed, and the astrocytes replaced by coarse glia fibers^7^.
**Perivascular sclerosis of the cerebral cortex** (*perivasculäre Sklerose* *der Hirnrinde*). Seemingly a related condition to the previous subform which, depending on the affected site, can resemble clinically Senile dementia (e.g., Perivascular gliosis of the frontal lobe), or a slowly developing focal brain disease (cortical paralysis, aphasic symptoms, cortical deafness or blindness). Gross examination of the gyri proves unremarkable, where the severity of the cortical changes can be easily overlooked^7^.
**Senile sclerosis of the cerebral cortex** (*senile Sklerose der Hirnrinde*). The cerebral cortex exclusively affected due to arteriosclerotic degeneration of the small cortical vessels, in its pure form, along with sclerosis of small cortical areas with loss of the nervous elements and proliferation of sustaining tissue [glia]. Old foci often with wedgeshaped form, the wide base oriented toward the cortical surface^8^.
**Senile cortical sclerosis [atrophy]** (*senile Rindeverödung*). Due to disease of the short vessels from the pia mater entering the cerebral cortex, causing small wedge-shaped foci in the superficial cortex, with the base oriented toward the cortical surface, besides deeper cortical foci. Destroyed ganglion cells and myelinated fibers replaced by a dense glia felt [gliosis]. Foci often situated in clusters, in the supply territory of a larger artery, the more superficial related to punctate retractions on the cortical surface^9^.

**Box 2:** Subcortical atrophy group, comprising subforms described and named by Alzheimer and Binswanger (condensed excerpts).

**Arteriosclerotic brain degeneration** (*arteriosklerotische Hirndegeneration*). Described by both Alzheimer and Binswanger, macro- and microscopically^7^. Course of months or years, with periodic attacks, progressing to severe dementia. Reminiscent personality, insight and judgement, ordered behavior, consciousness of disease, maintained until later stages (as opposed to Paralysis [neurolues]). Neurologic signs (absence or late appearance of pupillary rigidity, slowing of speech, cephalic, truncal and appendicular paresis, hemiparesis, and following attacks, ataxia and aphasia) helpful for diagnosis^7^. Brain weight loss, ventricular enlargement, widened vessel holes [perivascular spaces], discoloration and atrophy of adjacent tissue, especially of the basal ganglia and internal capsule, on gross examination. Microscopy with focal changes in the white matter and cortex, degeneration and loss of numerous ganglion cells; myelinated cortical fibers (tangential layer), radiations and deep white matter greatly decreased. Alzheimer indicated diseased long vessels of the white matter, with resulting secondary medullary [white matter] degeneration, as the main cause^7,8^.
**Chronic progressive subcortical encephalitis** (*Encephalitis subcorticalis chronica progressiva*). Described by Binswanger (1894), and named after him by Alzheimer (1902). Binswanger’s text partially quoted by Alzheimer (1898)^7^, emphasizing prominent atrophy of the white matter attributed to nourishment disturbance due to severe arteriosclerosis, affecting one or several gyri, and when marked, an entire brain lobe, preferentially of posterior brain slices, with inferior and posterior horn enlargement, on macroscopy^7^. Theme revisited by Alzheimer (1902), with some divergent gross findings, and addition of microscopic examination^9^. Mildly narrowed, but deeply sunken gyri, well preserved cortex, shrunken and discolored white matter, enlarged ventricles, on gross neuropathology. Scarcely affected cortex (Nissl preparations), even in advanced stages, deep white matter lacking or highly pale in many places, with gyral core white matter, and short subcortical association fibers preserved, on microscopy. Alzheimer indicated severe arteriosclerosis of the long vessels of the deep white matter, causing the highest atrophic changes, as the underlying cause^9^. Clinical course, according to Binswanger, as quoted by Alzheimer, characterized by slow decline of mental strength, difficulty and finally loss of the associative linking between certain cortical sensory areas or motor regions, with varied symptoms (hemianopia, hemiparesis with sensory loss, decline of the intellect). Protracted evolution, and in the terminal stage the patients becoming comparable to brainless experimental animals^7^. Later, Alzheimer added the emergence of a difficulty of association of ideas, followed by gradual language deficits, and often linguistic association difficulties. Spells (dizziness, epileptic, apoplectic), with related symptoms (visual field constriction, asymbolia, sensory and motor aphasia, agraphia, monoparesis, hemiparesis, among others) might occur, often disappearing, but tending to recur and remain stable; consciousness of disease maintained for a long period, despite deep mental decay; appearance of severe dementia, lasting for years, or progressing to death within months^9^.
**Arteriosclerotic atrophy of hemispheric white matter** (*arteriosklerotische Atrophie des Hemisphärenmarkes*). Alzheimer presented and named a short account of this subform. The cause lay in arteriosclerotic degeneration of the long vessels of the white matter, associated with loss of myelinated fibers, and vanished white matter replaced by glial proliferation. White matter atrophy could eventually reach severe degrees, the cerebral cortex, in pure cases, being only secondarily affected^8^.

**Box 3:** The apoplectic group, comprising a subform according to Alzheimer (condensed excerpts).

**Apoplectic dementia** (*Dementia apoplectica*) or **Post-apoplectic dementia** (*Dementia post apoplexiam*). According to Alzheimer (1898), this subform initially called Apoplectic dementia^7^ was attributed to a focal occurrence (apoplexy), subsequently evolving to a slowly progressive dementia resembling that found among the aged (*Greisenblödsinns*). He attributed the anatomical underpinning to cortical changes, also including the hemisphere not affected by the apoplexy. Alzheimer (1902) returned to the topic, indicating the basal ganglia and internal capsule region as preferential sites of atheromatosis and hemorrhages. A peculiar disease picture, which he designated Post-apoplectic dementia at the time, often evolved. However, microscopy revealed that dementia should be ascribed to hemispheric arteriosclerotic foci, and not to the apoplexy alone. This assumption was supported, besides the histological findings, by evidence that dementia was already perceptible before the apoplexy^9^. Concerning the clinical aspects, he based his account on Beyer’s (1896), who had described the mental state in Apoplectic dementia, characterized by apathy, labile mood, blunting to external world occurrences, orientation deficiency, confabulation, memory weakness for recent past with good recollection for remoter past issues. Also often associated with slower and sluggish speech, one-sided symptoms, among other changes^7^.

## COMMENTARIES

Here, the main features of the above-described condensed excerpts of Alzheimer's subforms will be commented upon, pathophysiological correlates provided, and some of the findings, in contemporary terms, interpreted.

**The gliosis and sclerosis group.** This group includes focal or diffuse vascular subforms, according to the lesion spread. In all subforms of this group, severe arteriosclerosis and glial proliferation constitute the neuropathological hallmark features, the degenerated vessels producing nervous tissue injuries. The lesions (foci) may have subcortical and cortical distribution as in the first two subforms, and subcortical distribution and/or cortical wedge-shaped lesions in the remaining ones. All display small or punctate retractions and mild or coarse granulation on the cortical surface, being circumscribed or extensive and diffuse, according to the underlying pathology. The difference among these subforms has more of a quantitative than a qualitative nature. As expected, the clinical manifestations depend on the location and extent of the neuropathological injuries. The retractions and granulated cortical surface changes, described in the subforms featuring superficial lesions, were interpreted by Román as consistent with "granular atrophy of the cerebral cortex".^10^ The subcortical foci appear to fit better, in present day terms, with "small infarcts" or with "microinfarcts",^11^ while the cortical wedge-shaped foci were more recently identified in autopsy studies as "cortical microinfarcts", described as minute foci with neuronal loss, gliosis, pallor, or more cystic lesions, considered an important neuropathological correlate of cognitive impairment and a contributor to dementia development, which escapes detection by conventional magnetic resonance imaging due to their small size. They are found in all brain regions, possibly more so in the cerebral cortex.^13-16^

**Subcortical atrophy group.** This group includes mainly diffuse vascular subforms. Alzheimer^8,9^ considered that the Arteriosclerotic brain degeneration (described by Binswanger and Alzheimer) differed in essence only in degree compared to the Arteriosclerotic atrophy of hemispheric white matter (described by Alzheimer), and Binswanger's Chronic progressive subcortical encephalitis. He emphasized that the main cause of the disease, in all three conditions, was the severe arteriosclerotic diseases of the long vessels of the deep white matter [penetrating arteries], with its resultant secondary degeneration. However, such extensive damage of white matter as seen in Binswanger's subform, is only rarely reached in the other forms. Thus, Alzheimer apparently suggested that these three subforms represented a spectrum differing only in a quantitative manner in relation to the extent of the white matter lesions.^8,9^

Binswanger's original description^17^ was quoted partially by Alzheimer (1898). Later, Alzheimer described the subform thoroughly (1902), possibly based on his own material, presenting a somewhat different macroscopic account, and adding the missing microscopic findings. Nonetheless, both emphasized the extensive deep white matter degeneration, sparing of the cortical intrinsic myelinated fibers, the gyral core white matter, and the immediately subcortical short association fibers [U fibers], in typical cases. Both stressed a loss of the associative linking between particular cortical sensory and motor areas, responsible for some of the clinical manifestations.^7,9,17^ It must be emphasized that Binswanger's description was based on an atypical case.^18,19^ Despite the atypicality, Binswanger's observation was insightful, with a detailed description of the course of the case, followed for almost ten years, summarized by the main symptoms (initial motor aphasia, followed by paraphasia, dysgraphia, paralexia and dyslexia, memory deficit, among others). Binswanger's macroscopic description revealed a severe loss of white matter in the parietal and temporal lobes, especially on the left side, and severe atrophy of the frontal cortex. The progress of the disease corroborated the pattern of white matter loss, particularly the conduction pathways between language centers and those of related functions (object images, acoustic language, reading and writing, and motor language centers). According to this destruction of associative fibers, the aphasic symptoms comprised characteristics of transcortical and intercentral conduction aphasia (conduction aphasia, Wernicke's *Leitungsaphasie*), besides other symptoms, as claimed by Binswanger. The further evolution of the ailment clearly indicated that the fiber loss was not restricted only to the brain regions cited. The final mental decay indicated that the entire hemispheric white matter was affected, bilaterally, with damage and destruction of numerous pathways.^17^ This description reveals the complex connections of the language centers, and the resultant symptoms compatible with the language-related disconnection syndromes studied today,^20-23^ highly frequent in vascular brain diseases.

This subform suffered numerous criticisms over time,^24^ but despite these the condition survived as a disease entity until the present day, maintaining the designation given by Alzheimer,^9^ and later ratified by several authors.^11,24,25^ It is noteworthy that Durand Fardel (1854) had previously described a similar condition he named *atrophie interstitielle du cerveau* (interstitial atrophy of the brain).^12^

Considering the quantitative differences among the subcortical subforms, already acknowledged by Alzheimer,^9^ probably all of the group merged with Binswanger's, to give the surviving one. Further details on the condition, as described by Binswanger, already partially presented,^18^ will be reviewed at a later date.

In present day terms, these subforms appear to represent the "Subcortical Ischemic Vascular Disease", having Binswanger's disease as the clearest expression.^11,26^

**The apoplectic group.** This group represents mainly focal disorders, at least in the beginning. Followed, in cases that evolve to dementia, by the appearance of more diffuse manifestations. Apoplexies are of longstanding familiarity, dating back to the pioneer studies of Johann Jakob Wepfer (1620-1695) in *Historiae apoplecticorum* (1658), and of Thomas Willis (1621-1675), who described Post-apoplexy dementia in *De Anima Brutorum* (1672). Later, several works appeared, most notably Durand-Fardel's *Traitée du Ramolissement du Cérveau* (1843).^12,27^

Binswanger, in 1894, had already made a brief comment on the clinical features of Post-apoplectic dementia.^17^ Alzheimer's contribution^9^ was in the form of microscopic description of these lesions, borrowing the clinical account from Beyer (1896). He attributed the cause to hemispheric arteriosclerotic foci, and not only to apoplexy alone. Additionally, he stated that this assumption was corroborated by dementia symptoms prior to the apoplectic episode. This early observation on the issue was, much later, verified many times.^28^

Summing up, the above-presented subforms of atheromatous vascular degeneration of the brain arteries studied by Alzheimer, as a suggestion here divided into 3 groups, appear to be related to neuropathological as well as clinical, cognitive and behavioral manifestations, which provided the basis, together with part of the main vascular forms presented in earlier papers, for the contemporary condition designated "Vascular Cognitive Impairment", with its varied underlying neuropathological correlates and clinical nuances.^29,30^
